# Supplementary material for: Coupling between Ribotypic and Phenotypic Traits of Protists across Life Cycle Stages and Temperatures
Source: Microbiol Spectr. 2021 Nov 24;9(3):e01738-21. doi: 10.1128/Spectrum.01738-21 (PMC8612162; doi:10.1128/Spectrum.01738-21)
Supplement: SUPPLEMENTAL FILE 1 — Supplemental material. Download SPECTRUM01738-21_Supp_1_seq11.pdf, PDF file, 0.7 MB [file spectrum01738-21_supp_1_seq11.pdf]

Supporting information

Figure S1. Standard curves of quantitative PCR in determining single-cell rDNA or rRNA (cDNA) copy numbers in *Colpoda* spp. The values of R^2 (0.998 ~ 1.000) and amplification efficiencies (E, 98% ~ 108.9%) are shown.

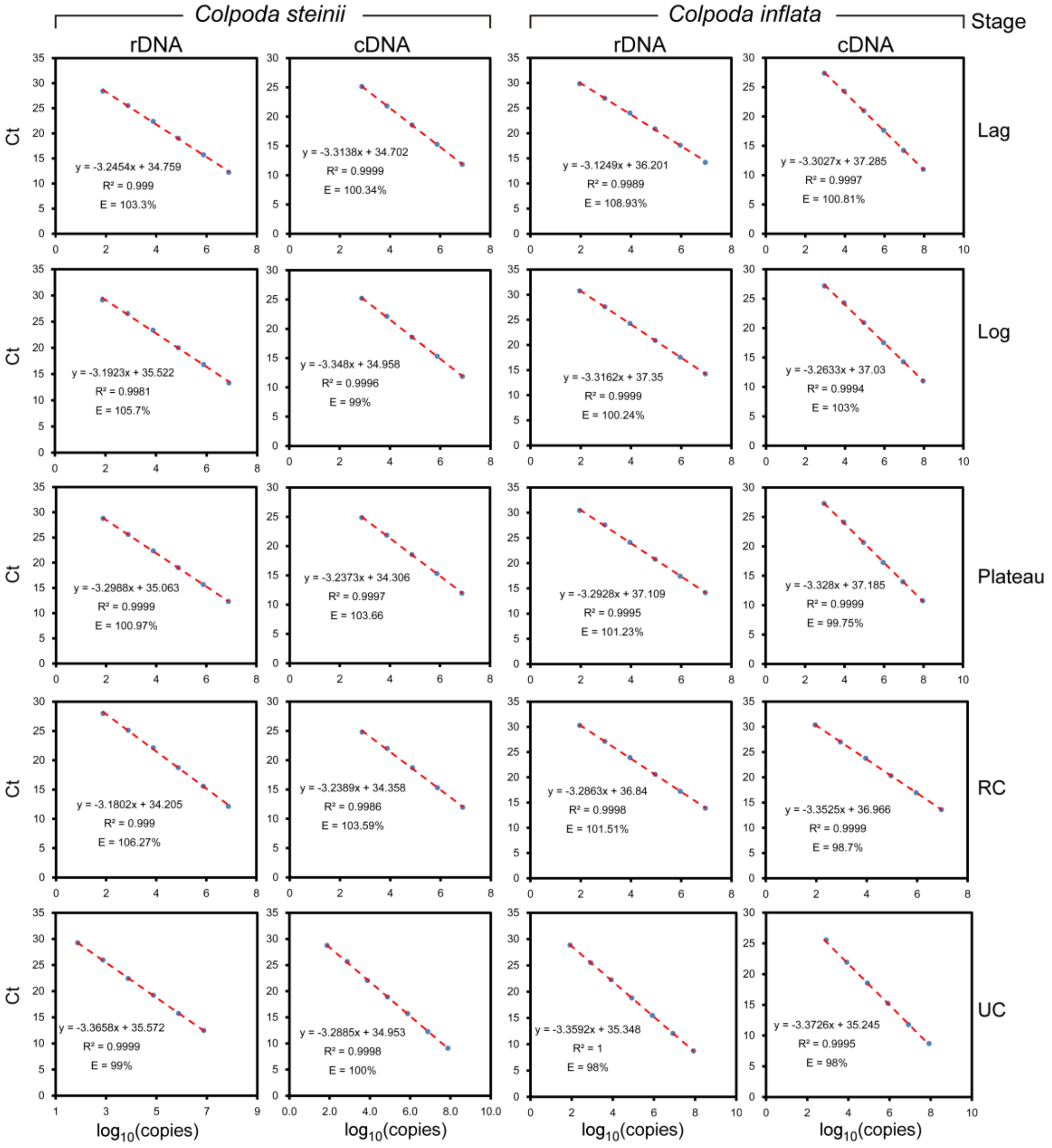

Figure S2. Electrophoretogram of PCR products obtained via amplification of 35 cycles of rDNA fragment. The single-cell nucleic acids (containing both rDNA and rRNA) were treated with different amounts of DNase (0 to 3 μL) for various periods of time (10, 30, and 60 min). There were no detectable target bands of rDNA fragments in the samples which were treated with 1 μL (2 U) of DNase for 60 min (lanes 5 and 8), indicating optimal incubation conditions for complete degradation of rDNA. M, DNA markers. Lane 12 indicates negative control with double-distill water.

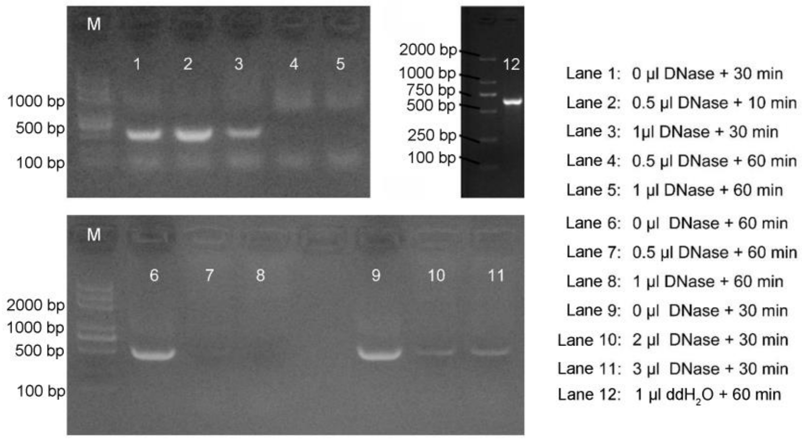

Figure S3. Chilling treatments of two *Colpoda* species induced unstable cyst formation. The chilling treatments at 0 ℃ and 4 ℃ showed that cold shock could induce formation of unstable cysts in actively growing *Colpoda* cells. Nevertheless, this effect was strongly dependent on chilling temperature and duration. Putting both *Colpoda* species at 10 ℃ did not induce formation of unstable cysts (data not shown). **(A)** Cell division of *Colpoda steinii* was completely inhibited upon ice water chilling, and nearly 100% of vegetative cells were transformed into unstable cysts in 4 hrs. Longer chilling duration led to a decrease in the number of unstable cysts. **(B)** In contrast, the population of *C. inflata* collapsed completely during ice water treatments within 3 hrs. **(C)** At 4 ℃, the small-sized *C. steinii* continued to grow and divide during the first 1.5 hrs. Afterwards, total cell abundance (= vegetative cells + unstable cysts) remained relatively stable, with approximately 76% of cells being transformed from vegetative status into unstable cysts following incubation for 9 to 12 hrs. Treating cells at 4 ℃ for a longer period of time damaged both types of cell. **(D)** Chilling at 4 ℃ completely inhibited cell division of *C. inflata*; unstable cysts progressively increased to 37% in 10 hrs, after which the number of unstable cysts remained stable for the next 8 hrs. All individuals transformed into unstable cysts in 20 hrs. Approximately, 36% of cells were lost during this incubation.

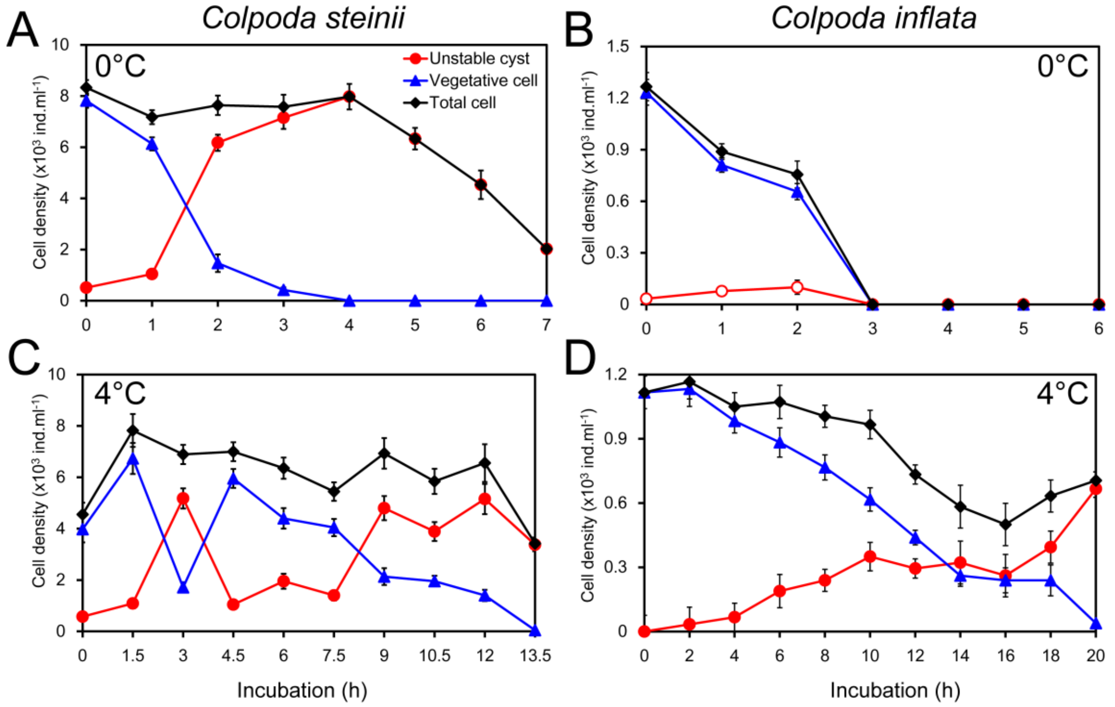

Figure S4. The per-cell rDNA and rRNA copy numbers (CNs) in two *Colpoda* species across life-cycle stages (lag, log, plateau phases and resting cysts) was significantly related to macronuclear volume. The rRNA CNs in RC appeared to be much lower than similarly sized vegetative cells or unstable cysts, such that the coefficients of determination (R^2) of regressions were much lower with resting cysts being taken into account (+RC) than without (-RC). RC = resting cyst.

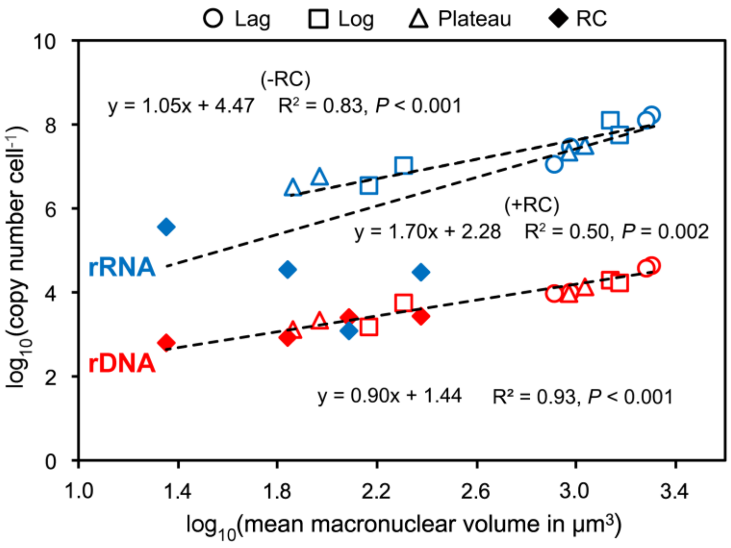

Figure S5. Regressions between rDNA and rRNA-based OTU numbers and cell volume.

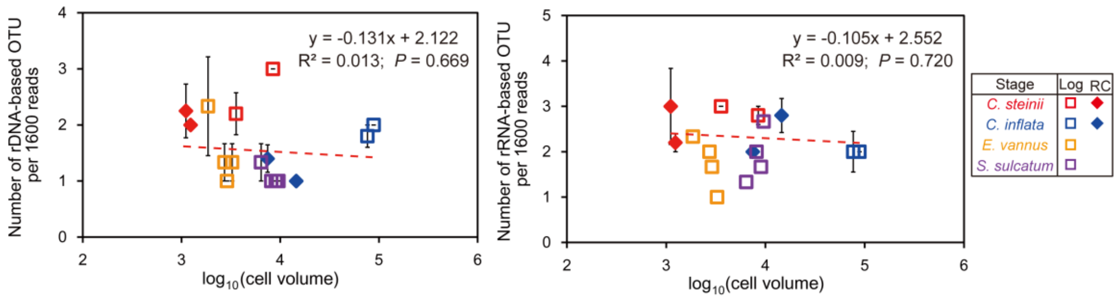

Table S1. Numbers of reads (mean ± standard error) obtained at processing steps and resulting amplicon sequence variants (ASVs).

Species name	Life stage	Ribotype	Temp (℃)	Raw seqs	Filtered seqs	Merged seqs	Nonchimeras seqs	Retained reads (%)*	Range of OTU number# (All reads-based)	OTU number# (All reads-based)	OTU number# (Rarefied at 1600 reads)	*n*
Colpoda steinii	RC	DNA	18	91999 ± 5186	78718 ± 3759	78160 ± 3689	77837 ± 3616	85 ± 2	2 ~ 3	2.3 ± 0.3	2.0 ± 0.0	3
			28	96381 ± 1323	85225 ± 2575	84805 ± 2674	84599 ± 2767	88 ± 2	1 ~ 4	2.5 ± 0.6	2.2 ± 0.1	4
		RNA	18	89501 ± 3104	77448 ± 3135	76601 ± 3235	75742 ± 3505	85 ± 2	2 ~ 5	3.4 ± 0.5	2.0 ± 0.0	5
			28	91410 ± 2446	80311 ± 2601	79006 ± 2836	78873 ± 2833	86 ± 1	2 ~ 5	3.8 ± 0.6	2.9 ± 0.1	5
	Log	DNA	18	92836 ± 2137	81968 ± 1847	81630 ± 1843	81457 ± 1831	88 ± 0	3 ~ 3	3.0 ± 0.0	2.1 ± 0.0	5
			28	91359 ± 3405	80880 ± 3116	80600 ± 3130	80420 ± 3119	88 ± 1	1 ~ 4	2.8 ± 0.5	2.0 ± 0.0	5
		RNA	18	86413 ± 1777	76451 ± 1612	75981 ± 1608	75854 ± 1591	88 ± 0	3 ~ 4	3.2 ± 0.2	2.1 ± 0.0	5
			28	90017 ± 3066	79539 ± 2526	78948 ± 2540	78623 ± 2555	87 ± 0	3 ~ 4	3.2 ± 0.2	2.1 ± 0.0	5
Colpoda inflata	RC	DNA	18	89468 ± 2956	78149 ± 2729	77865 ± 2730	77625 ± 2758	87 ± 1	1 ~ 2	1.2 ± 0.2	1.0 ± 0.0	5
			28	87593 ± 2039	75883 ± 1826	75366 ± 1893	74732 ± 1825	85 ± 0	1 ~ 3	1.6 ± 0.4	1.3 ± 0.0	5
		RNA	18	92588 ± 2103	81082 ± 2102	80174 ± 2058	78524 ± 2138	85 ± 1	2 ~ 4	3.6 ± 0.4	2.3 ± 0.1	5
			28	90218 ± 3277	78868 ± 2550	77950 ± 2530	77067 ± 2511	85 ± 0	2 ~ 4	3.0 ± 0.3	2.0 ± 0.0	5
	Log	DNA	18	91796 ± 3184	82520 ± 2623	82312 ± 2663	82175 ± 2638	90 ± 0	2 ~ 2	2.0 ± 0.0	1.1 ± 0.0	4
			28	87413 ± 2711	78497 ± 2547	78186 ± 2558	78125 ± 2567	89 ± 0	1 ~ 4	2.4 ± 0.5	1.0 ± 0.0	5
		RNA	18	90949 ± 2536	82514 ± 2489	82155 ± 2476	82095 ± 2482	90 ± 0	2 ~ 4	2.8 ± 0.5	1.1 ± 0.0	5
			28	93911 ± 2019	84351 ± 1635	84003 ± 1646	83959 ± 1631	89 ± 0	3 ~ 4	3.4 ± 0.2	1.1 ± 0.0	5
Euplotes vannus	Log	DNA	16	43540 ± 593	39011 ± 1425	38959 ± 1444	38926 ± 1457	90 ± 5	2 ~ 2	2.0 ± 0.0	1.0 ± 0.0	3
		RNA		43962 ± 6948	39696 ± 7819	39655 ± 7810	39651 ± 7809	89 ± 5	2 ~ 4	3.0 ± 0.6	1.4 ± 0.1	3
		DNA	21	40096 ± 2735	35967 ± 4133	35934 ± 4135	35919 ± 4137	89 ± 5	2 ~ 4	3.0 ± 0.6	1.0 ± 0.0	3
		RNA		42861 ± 7676	38305 ± 8531	38251 ± 8514	38251 ± 8514	88 ± 6	1 ~ 4	3.0 ± 1.0	1.3 ± 0.1	3

		DNA	25	46696 ±1993	41700 ±3110	41639 ±3122	41628 ±3122	89 ±5	3 ~ 5	4.0 ±0.6	1.3 ±0.1	3
		RNA		49308 ±3910	44410 ±5968	44346 ±5968	44345 ±5969	89 ±5	1 ~ 6	3.3 ±1.5	1.3 ±0.1	3
		DNA	16*	38691 ±3621	34395 ±2613	34361 ±2604	34360 ±2603	89 ±5	2 ~ 3	2.7 ±0.3	1.0 ±0.0	3
		RNA		43468 ±1238	38958 ±1616	38906 ±1624	38904 ±1623	90 ±4	3 ~ 4	3.7 ±0.3	2.3 ±0.1	3
Strombidiu	Log	DNA	16	40614 ±4207	33479 ±3249	33424 ±3236	33316 ±3127	82 ±1	2 ~ 3	2.5 ±0.5	1.0 ±0.0	2
m sulcatum		RNA		46633 ±3220	41632 ±5145	41559 ±5173	41559 ±5173	88 ±5	3 ~ 5	4.3 ±0.7	2.0 ±0.2	3
		DNA	21	37720 ±3699	32927 ±3997	32873 ±4021	32778 ±4115	87 ±6	2 ~ 5	3.0 ±1.0	1.3 ±0.1	3
		RNA		42470 ±3289	37398 ±3879	37322 ±3873	37313 ±3882	88 ±5	3 ~ 5	4.0 ±0.6	2.0 ±0.1	3
		DNA	25	42498 ±1378	37617 ±3237	37572 ±3247	37568 ±3248	88 ±5	2 ~ 6	4.0 ±1.2	2.2 ±0.1	3
		RNA		44956 ±5466	40001 ±7350	39924 ±7377	39917 ±7379	88 ±5	2 ~ 4	3.0 ±0.6	2.3 ±0.1	3
		DNA	16*	39090 ±1679	34395 ±1670	34334 ±1670	34319 ±1667	88 ±4	2 ~ 4	3.0 ±0.6	1.3 ±0.1	3
		RNA		44310 ±3017	38509 ±3954	38445 ±3935	38445 ±3935	86 ±3	2 ~ 3	2.3 ±0.3	1.0 ±0.0	3

* Retained reads (%) indicate the proportion of retained reads with no chimeras to the number of raw reads of a sample.

[#], the OTUs are defined at a sequence similarity cutoff of 99% for *C. steinii* and *C. inflata*, and at 100% for *E. vannus* and *S. sulcatum*, based on the results of sequencing and analysis of individual clones with 18S rDNA or cDNA fragments.

n, the number of biological replicates.

Table S2. Performance of the DADA2 workflow in processing throughput sequencing data of randomly selected five individual clones inserted with 18S rDNA or cDNA (rRNA) fragments of four ciliate species. The numbers of unique amplicon sequence variants (ASVs, the reads that are 100% identical) and operational taxonomic units (OTUs) defined at a series of similarity threshold ranging from 99% to 90% are shown.

**Species**	**Sample ID**	**ASV**	**OTU**									
		**100**	**99**	**98**	**97**	**96**	**95**	**94**	**93**	**92**	**91**	**90**
C. inflata	CI1	12	1	1	1	1	1	1	1	1	1	1
	CI2	12	1	1	1	1	1	1	1	1	1	1
	CI3	12	1	1	1	1	1	1	1	1	1	1
	CI4	12	1	1	1	1	1	1	1	1	1	1
	CI5	12	1	1	1	1	1	1	1	1	1	1
C. steinii	CS1	12	1	1	1	1	1	1	1	1	1	1
	CS2	12	1	1	1	1	1	1	1	1	1	1
	CS3	12	1	1	1	1	1	1	1	1	1	1
	CS4	12	1	1	1	1	1	1	1	1	1	1
	CS5	12	1	1	1	1	1	1	1	1	1	1
E. vannus	EV1	1	1	1	1	1	1	1	1	1	1	1
	EV2	1	1	1	1	1	1	1	1	1	1	1
	EV3	1	1	1	1	1	1	1	1	1	1	1
	EV4	1	1	1	1	1	1	1	1	1	1	1
	EV5	1	1	1	1	1	1	1	1	1	1	1
S. sulcatum	SS1	1	1	1	1	1	1	1	1	1	1	1
	SS2	1	1	1	1	1	1	1	1	1	1	1
	SS3	1	1	1	1	1	1	1	1	1	1	1
	SS4	1	1	1	1	1	1	1	1	1	1	1
	SS5	1	1	1	1	1	1	1	1	1	1	1
